# C-reactive protein: a target for therapy to reduce inflammation

**DOI:** 10.3389/fimmu.2023.1237729

**Published:** 2023-07-26

**Authors:** Salma A. Rizo-Téllez, Meriem Sekheri, János G. Filep

**Affiliations:** ^1^ Department of Pathology and Cell Biology, University of Montreal, Montreal, QC, Canada; ^2^ Research Center, Maisonneuve-Rosemont Hospital, Montreal, QC, Canada

**Keywords:** C-reactive protein, monomeric CRP, CRP antagonists, CRP lowering therapies, inflammation, cardiovascular disease, autoimmunity, cancer

## Abstract

C-reactive protein (CRP) is well-recognized as a sensitive biomarker of inflammation. Association of elevations in plasma/serum CRP level with disease state has received considerable attention, even though CRP is not a specific indicator of a single disease state. Circulating CRP levels have been monitored with a varying degree of success to gauge disease severity or to predict disease progression and outcome. Elevations in CRP level have been implicated as a useful marker to identify patients at risk for cardiovascular disease and certain cancers, and to guide therapy in a context-dependent manner. Since even strong associations do not establish causality, the pathogenic role of CRP has often been over-interpreted. CRP functions as an important modulator of host defense against bacterial infection, tissue injury and autoimmunity. CRP exists in conformationally distinct forms, which exhibit distinct functional properties and help explaining the diverse, often contradictory effects attributed to CRP. In particular, dissociation of native pentameric CRP into its subunits, monomeric CRP, unmasks “hidden” pro-inflammatory activities in pentameric CRP. Here, we review recent advances in CRP targeting strategies, therapeutic lowering of circulating CRP level and development of CRP antagonists, and a conformation change inhibitor in particular. We will also discuss their therapeutic potential in mitigating the deleterious actions attributed to CRP under various pathologies, including cardiovascular, pulmonary and autoimmune diseases and cancer.

## Introduction

The prototypic acute-phase reactant C-reactive protein (CRP), discovered as a protein that precipitates C-polysaccharide of *Streptococcus pneumonia*e, has long been recognized as a sensitive biomarker of inflammation (1). Elevations in baseline serum CRP level have been detected in numerous pathologies, and have been suggested to being useful to monitor disease progression. CRP has received considerable attention as a diagnostic and prognostic marker in autoimmune diseases (2, 3), cardiovascular diseases (4–6), chronic kidney disease (7), cancer (8) and COVID-19 (9, 10) as well as for guiding therapy (8, 11). Although there is a continuing debate over whether CRP is primarily a passive indicator of inflammation or is a “culprit” mediating disease (12–18), CRP plays important roles in host defense against invading pathogens, autoimmunity and inflammation. CRP exhibits many, often conflicting pro- and anti-inflammatory activities (14, 19–21), which makes delineating its pathogenetic roles even more challenging. Results from structure-function studies challenge the long-held and rather simplistic view of CRP as a stable pentameric protein and identified conformationally distinct forms, including native pentameric CRP (pCRP) and modified/monomeric CRP (mCRP), which exhibit distinct functional properties and may explain many of the opposing biological activities attributed to CRP (21, 22). CRP synthesis, structure and biological activities have been reviewed in detail elsewhere (19, 21, 23–25). In this review, we aimed to critically assess the competing views on the role of CRP isomers in disease pathogenesis and therapy, focusing on recent advances that may provide a rationale basis for guiding therapy and/or therapeutic targeting of CRP isomers to limit inflammation underlying various diseases.

## CRP expression, structural properties

Native CRP is member of the pentraxin family, an evolutionarily highly conserved class of pattern recognition molecules. The human CRP gene, located on chromosome 1, q23-q24 encodes for a CRP subunit of a single polypeptide chain of 206 amino acids (23). CRP is composed of five identical non-covalently bound subunits forming a planar ring with a central pore (23). The two opposite faces of the pentamer, and thus each protomer, have distinct binding properties. The A-face binds and activates complement C1q, whereas the B-face contains the Ca^2+^-dependent binding pocket for phosphocholine, expressed by bacterial, fungal and eukaryotic cells (24, 26). CRP also binds to nuclear antigens, the oxidized LDL receptor, apoptotic cell membrane, glycan components of microorganisms, and many other ligands (24, 27, 28), though some of these interactions have been disputed.

Native CRP is predominantly synthesized in hepatocytes under transcriptional control by cytokines (IL-6 and to a lesser extent IL-1β and TNF-α), the transcription factors hepatic nuclear factor (HNF) 1α and HNF3 as part of the “reorchestration” of hepatic gene expression in response to infection or tissue injury (19), promoter methylation and a distal enhancer (29). Hepatic secretion of pCRP accounts for rapid increases in serum pCRP levels during the acute-phase reaction. The serum half-life of pCRP is about 19 h under both physiological and pathological conditions (30), thus directly reflecting the rate of its hepatic synthesis. Ethnicity, sex and polymorphism in the apoliprotein E and CRP genes are known to influence baseline serum pCRP levels in humans (31, 32). CRP gene polymorphism influences gene expression and may predispose to systemic lupus erythematosus (31), but do not appear to be associated with increased risk for cardiovascular diseases (33, 34). Additionally, the kidney has been reported as a second site of pCRP formation in humans (35). Expression of CRP mRNA and pCRP synthesis has also been detected in the diseased vessel wall, coronary artery bypass grafts and neurons (35–37). The contribution of these sites to circulating pCRP remains to be investigated.

Under physiological conditions, pCRP appears to exist in a NaCl concentration-dependent pentamer-decamer equilibrium (38). Another form of CRP, characterized by multiple-size lettuce-like structures of about 300-500kDa, were detected in the serum of obese patients (39). The origin and pathological significance of these CRP forms are not known.

## Elevated plasma CRP levels

Although not specific for a single disease process, CRP is commonly used as a static measurement and CRP levels have been correlated with disease activity and to some degree, severity and prognosis in several diseases. CRP has been promoted as an independent predictor of cardiovascular events and metabolic syndrome (40–42), though the association is considerably weaker than previously thought (43, 44). The data from Mendelian randomization studies (33, 34) coupled with animal studies with injection of human pCRP and transgenic mice over-expressing human CRP may support an association between CRP and cardiovascular disease, but provide no direct evidence for a causative role for pCRP. In the Dallas Heart study hs-CRP was not independently associated with atherosclerotic burden in the coronary artery and abdominal aorta (45), whereas the REVERSAL trial reported that lower hs-CRP levels were independently and significantly correlated with atherosclerosis progression (46). Other studies have concluded that hs-CRP likely serves as a biomarker of vascular inflammation underlying atherosclerosis (14, 44). Thus, whereas the potential of therapeutic targeting of pCRP in cardiovascular disease remains unresolved, hs-CRP has clinical usefulness in guiding therapy as discussed below.

Other clinical studies reported positive correlation between elevated plasma CRP levels and myocardial infarct size (47), reduced lung function in chronic obstructive pulmonary disease (48) or the severity of COVID-19-evoked respiratory distress (49, 50). Higher plasma CRP levels were found to predict flares in systemic lupus erythematosus (2) and to portend poor prognosis in melanoma (51). Patients with non-small cell lung cancer who received the immune checkpoint PD-1 inhibitor nivolumab, early increases in hs-CRP and IL-6 were predictive for the efficacy of treatment (52). Nivolumab evoked a CRP flare-response (defined as a rapid, more than twofold increase in CRP levels followed by decrease below baseline values within 3 months) in about 25% of patients with metastatic renal cell carcinoma and this was associated with significant tumor shrinkage and improved 1-year survival rate (53).

The association of CRP with prognosis should, however, be interpreted with caution as an indication of direct causal contribution of CRP to disease pathogenesis. A definitive way to test this is the use of compounds that specifically block binding of CRP to its ligands and/or receptors to assess its pro-inflammatory effects *in vivo*.

## Modulation of the inflammatory response by CRP isomers

**Figure 1:**
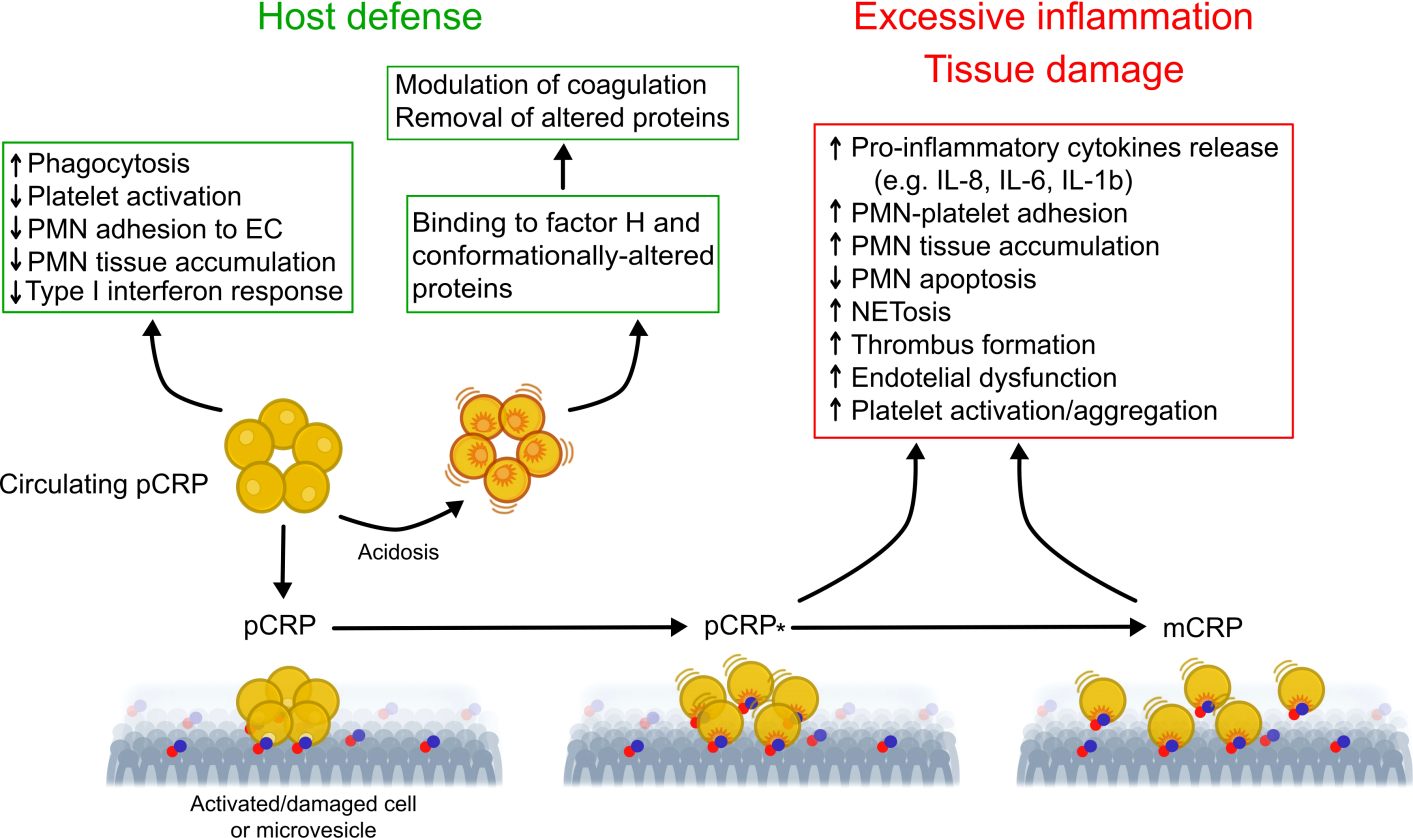
Proposed model for regulation of the inflammatory response by conformationally distinct CRP isomers. In the circulation, native CRP exists in a disc-shaped pentameric form (pCRP) and exhibits predominantly anti-inflammatory activities (e.g. opsonization) that are critical for clearance of invading bacteria and damaged cells by phagocytes. Thus, pCRP may limit further damage and prevent autoimmunity. Mild acidosis within the inflammatory locus unmasks additional binding sites in pCRP for complement factor H, which modulate coagulation and for conformationally altered proteins, thereby promoting their removal. Binding of pCRP to phosphocholine or phosphoethanolamine head groups exposed on the surface of activated or damaged cells, or microvesicles, leads to formation of a partially dissociated pentamer (pCRP*) followed by dissociation into its monomeric subunits (mCRP). Unlike pCRP, pCRP*/mCRP exhibit potent pro-inflammatory actions, including stimulation of thrombus formation, activation of endothelial cells, monocytes, platelets and neutrophils, neutrophil and monocyte adhesion to endothelial cells, enhanced formation of neutrophil-platelet and platelet-monocyte aggregates, production of pro-inflammatory cytokines IL-1β and IL-6, and extrusion of neutrophil extracellular traps (NET). These may contribute to excessive, non-resolving inflammation and to aggravation of tissue injury. This multistep mechanism would uncouple the local effects of pCRP*/mCRP from those of circulating pCRP, therefore contribute to localization of inflammation. Of note, microvesicle-associtaed pCRP*/mCRP may contribute spreading the inflammatory reaction to distant sites. EC, endothelial cells; mCRP, monomeric CRP; NETosis, extrusion of neutrophil extracellular traps; pCRP, pentameric CRP; pCRP*, partially dissociated pentameric CRP; PMN, polymorphonuclear neutrophil granulocytes. Modified from Wu et al. (21) and Filep (54).

While pCRP has been postulated to be stable under physiological conditions (24), compelling evidence indicates that CRP exists in conformationally distinct forms and conformational changes in pCRP results in expression of potent pro-inflammatory activities (**Figure 1**) (21). The mild acidic environment within inflamed tissues confers pCRP binding specificities for factor H (55) and conformationally altered proteins, such as oxidized LDL and complement C3b (56) that do not bind to pCRP at physiological pH. Binding of circulating pCRP to phosphocholine or phosphoethanolamine head groups of membrane lipids expressed on the surface of activated platelets or apoptotic cells induces the formation of a partially dissociated pentamer (pCRP*), which then dissociates into the monomeric subunits, mCRP (57–59). pCRP* and mCRP exhibit potent pro-inflammatory activities, including stimulation of IL-8 secretion from neutrophils and human coronary artery endothelial cells (60, 61), promote neutrophil adhesion to platelets and endothelial cells (62, 63), delay neutrophil apoptosis (64) and trigger extrusion of neutrophil extracellular traps (65), characteristic features of the inflammatory response. pCRP* binds and activate complement C1q (66), which, in turn, can amplify pre-existing inflammation and tissue damage (21, 57, 67). Accumulation of pro-inflammatory CRP isoforms with in inflamed/injured but not in healthy tissues, and local expression of mCRP, for example within arteriosclerotic plaques (68) and in circulating microparticles (69, 70) would ensure localization of inflammation (57) and precipitate tissue injury (21).

While purified human pCRP itself does not evoke inflammation when injected into healthy individuals (71), it can amplify tissue injury in animal models, induce the expression of adhesion molecules and production of IL-6 and IL-8 (24, 72–75). Caution should be exercised in interpreting these observations because many of these effects can be attributed to contaminants (endotoxin or the preservative sodium azide) in commercial CRP preparations not to pCRP itself (13, 21). By contrast, other studies documented pCRP protection against the assembly of the terminal complement attack complex (27) and bacterial sepsis (76), reversal of proteinuria in autoimmune mice (77), and prevention of autoimmunity (78). Recently, pCRP was found to reduce immune complex-triggered type I interferon response, consistent with a protective action in systemic lupus erythematosus (79). This action was lost in mCRP, further highlighting the complexity of regulation of immune response by CRP isomers in autoimmune diseases.

CRP isomers bind to distinct receptors. Thus, pCRP binds primarily to the low affinity Ig receptor FcγRIIa (CD32) and to some extent to the high affinity IgG receptor FCγRI (CD64) on phagocytes and endothelial cells (21, 80), and αI_Ib_β_3_integrin on platelets (81), whereas mCRP binds to FcγRIIIb (CD64) on phagocytes and lipid rafts on human endothelial cells (82).

The concept of activation-induced conformational changes could explain why pCRP itself is not pro-inflammatory in the absence of infection or tissue injury. Conformational changes in pCRP, and generation of pCRP* and mCRP, would unmask “hidden” pro-inflammatory activities that may collectively amplify the initial inflammatory signal evoked by infection or tissue injury, leading to exacerbation of tissue damage and more severe disease (21, 73). However, further studies are needed to explore the clinical importance of mCRP or other CRP conformers.

## CRP as a target for therapy

### Therapeutic lowering of serum CRP

The association of persisting modest elevations in plasma CRP level (detected by high sensitivity assays) with chronic diseases has attracted considerable clinical interest and often contradictory interpretations. CRP is generally recognized as a biomarker of ongoing inflammation and to a varying degree as a predictor of clinical outcome. Although conclusive evidence for a causal role for pCRP (and/or CRP isomers) is still lacking, lowering pCRP levels is widely anticipated to reduce the adverse effects attributed to CRP. Indeed, several approaches have been developed and tested for lowering plasma pCRP level or countering CRP`s actions (**Table 1**).

**Table 1:** CRP targeting strategies.

Agent	Species	Disease/Model	Effect	Reference
CRP lowering approaches				
*Lifestyle changes*				
- Exercise - Diet - Weight loss - Vitamin supplementation - Smoking cessation	Human	Healthy Cardiovascular disease Metabolic syndrome	↓ CRP levels ↓ cardiovascular events ↓ body weight	(83–87)
*Medication-associated decreases in CRP level*				
Statins	Human	Cardiovascular disease	↓ CRP levels (15-60%) ↓ Cholesterol ↓ cardiovascular events	(88–92)
Bempedoic acid	Human	Cardiovascular disease	↓ CRP levels by 27% ↓ Total cholesterol by 15%	(93)
Rosiglitazone Pioglitazone Dipeptidyl peptidase 4 inhibitors	Human	Diabetes	↓ CRP levels	(94, 95)
Angiotensin-converting enzyme inhibitors	Human	Cardiovascular disease Metabolic syndrome	↓ CRP levels ↓ IL-6 levels	(94, 96)
*Antisense oligonucleotides*				
CRP-mRNA antisense oligonucleotides	Human	Primary hepatocytes	↓ CRP mRNA	(97)
	Mouse	CRP transgenic mice with collagen-induced arthritis	↓ human CRP levels ↑ arthritis clinical score	
	Rat	Acute myocardial infarction	↓ CRP levels by >60% ↑ heart function	(98)
	Human	Healthy Rheumatoid arthritis	↓ CRP levels No effect on arthritis clinical score	(99)
		Healthy Endotoxin challenge	↓ CRP level	(100)
		Atrial fibrillation	↓ CRP levels by 64% No effect on atrial fibrillation burden	(101)
CPR selective apheresis				
PentraSorb^®^	Human	ST-segment Elevation Myocardial Infarction	↓ CRP levels by 50% ↓ infarct size ↑ wound healing	(47, 49, 102)
		Severe COVID-19	↓ CRP levels by 50-90% ↓ mortality	(49, 103–105)
*Small-molecule CRP inhibitors*				
(1,6-bis (phosphocholine) -hexane)	Rat	Acute myocardial infarction	↓ mortality ↓ infarct size ↓ cardiac dysfunction	(72)
		LPS-inflamed cremasteric tissue	↓ CRP deposition ↓ leukocyte adhesion	(59, 67)
		Acute myocardial infarction	↓ CRP deposition ↓ leukocyte infiltration ↓ caspase 3 expression ↓ TNF-α and IL-6 expression	
		Renal ischemia- reperfusion	↓ lesions ↑ excretory function ↓ monocyte infiltration	(75)
	Human	THP-1 or Jurkat cell-derived microvesicles	↓ calcium-dependent binding	(59)
	Mouse	Lethal influenza virus infection	↓ mortality ↓ viral titers ↓ lung lesions ↓ inflammatory cells infiltration	(106)
Phosphonate compound C10M	Human	ADP-activated platelets	↓ CRP binding to platelets	(65)
		Monocytes	↓ platelet–monocyte aggregates ↓ monocyte adhesion	
		Endothelial cells	↓ ICAM-1 and VCAM-1 expression	
		Neutrophils	↓ CD11b expression ↓ ROS production ↓ NET formation No effect on phagocytosis	
	Rat	Renal ischemia- reperfusion	↓ CRP deposition ↑ excretory function ↓ monocyte infiltration	
		Acute hindlimb allograft rejection	↓ graft loss ↓ monocyte infiltration ↓ CRP deposition	

NET, neutrophil extracellular traps; ROS, reactive oxygen species. ↓ (arrow down) decreased. ↑ (arrow up) increased.

Observational studies reported a relationship between life style changes, encouraging cessation of smoking, weight loss, more physical activity and Mediterranean diet, with concurrent reduction in hs-CRP levels and the risk for future cardiovascular events (83–86). Studies with angiotensin converting enzyme inhibitors, angiotensin II (type I) receptor blockers, vitamins E and C, and the anti-platelet agents clopidogrel and aspirin, yielded conflicting results in regard with their efficiency to lowering hs-CRP levels (94, 107). Unlike insulin, the anti-diabetic drugs rosiglitazone and pioglitazone have been found to significantly decrease serum CRP, though the molecular mechanisms and the potential clinical benefits remain largely unknown (94).

Evidence derived mainly from trials with statins support the potential value of hs-CRP for primary and secondary prevention of cardiovascular disease, though this notion still remains controversial. The landmark JUPITER trial demonstrated the benefits of rosuvastatin therapy in primary prevention as well as the utility of hs-CRP for identifying a population at risk for cardiovascular disease (88–90). A new meta-analysis from the PROMINENT, REDUCE-IT and STRENGTH trials (which were originally designed to test triglyceride-lowering) showed that inflammation, and thus hs-CRP is more tightly linked than LDL cholesterol to future adverse effects in patients already on statins (91). Limitations of this analysis include the effects of confounding bias (e.g. high intensity statin use and diabetes) and lack of attention to primary versus secondary prevention (108). These concerns notwithstanding, the meta-analysis would argue for routine hs-CRP testing to assess residual inflammatory risk (109) and a combined approach to aggressive lipid-lowering and inflammation-inhibiting therapy with colchicine, IL-1 or IL-6 inhibitors or bempedoic acid (91, 93).

### Antisense oligonucleotides

Another approach to reduce CRP production is the use of antisense oligonucleotides (ASO) to specifically inhibit mRNA translation in particular in the liver, the predominant site of pCRP synthesis (23) where ASOs have a propensity to accumulate (110). Lowering plasma CRP level with rat-specific ASO ISIS 197178 was associated with reduction of infarct size and improved cardiac function in a rat model of myocardial infarction (111). Human-specific ASO ISIS 353512 reduced neointima formation in human CRP transgenic mice subjected to carotid artery ligation (98). In healthy subjects, ASO ISIS 329993 (ISIS-CRP_Rx_) reduced by about 70% of the peak plasma CRP response to endotoxin challenge without affecting cytokine production and coagulation (100). Treatment with ISIS-CRP_Rx_ of patients with rheumatoid arthritis also decreased plasma CRP level, but did not reduce disease activity (99). Unexpectedly, another ASO, ISIS 353512 increased IL-6 and CRP levels in healthy volunteers (112), illustrating the challenges with CRP-ASO therapy.

### Selective CRP apheresis

Another strategy to investigate pathogenetic roles for pCRP (and arguably other CRP isomers) is reducing plasma pCRP level by selective apheresis, which appears to be a relatively simple, efficient and clinically safe approach (49, 113). In this protocol, patients are subjected to a 4-6 h extracorporeal circuit and blood plasma is applied to phosphocholine-linked resin. A case report and studies on small cohorts of patients with ST elevation myocardial infarction (STEMI) demonstrated efficient lowering of plasma CRP levels (47, 102, 114, 115). However, results from the multi-center pilot CAMI-1 (CRP Apheresis in Acute Myocardial Infarction-1) study were inconclusive in regard with correlation of reduced CRP levels with myocardial infarct size (47) because the study was not randomized. The ongoing trials on the effects of CRP apheresis on the course of STEMI (NCT04939805) and ischemic stroke (CASTRO-B, NCT03884153) are anticipated to address this issue. There are several case reports with mixed results on reducing lung injury with CRP apheresis in patients with COVID-19-evoked respiratory distress syndrome (49, 103–105). The subsequently planned trial on pulmonary, myocardial and/or renal injury in COVID-19 (NCT04898062) has been withdrawn [https://www.clinical.trials.gov, as of June 4, 2023].

Similar to other CRP lowering strategies, the fundamental question whether the beneficial effects can be attributed to lowering pCRP level directly or to reduction of formation of conformationally altered CRP secondary to reduced availability of the parent molecule pCRP remains unanswered. While short-term reductions of plasma CRP levels may be beneficial under certain circumstances, markedly lower CRP levels over prolonged periods of time may impair antimicrobial defense, and thus augmenting the risk of bacterial or viral infection. Whether this would limit the clinical utility of CRP apheresis and what plasma CRP levels after CRP apheresis will be still sufficient to support innate defense functions remain to be investigated.

### Small molecule CRP inhibitors

Two distinct CRP targeting strategies have been developed. In 2006, the Pepys group has designed and synthesized the first small-molecule inhibitor of CRP (72). The bivalent compound, 1,6-bis(phosphocholine)-hexane (bis-PC) bridges the phosphocholine-binding pockets on the B-face of two separate CRP pentamers, bringing the phosphocholine-binding surfaces together in a parallel fashion (72). The resulting decamer structure stabilizes conformation of pCRP and prevents binding of other ligands to the B-face. Bis-PC has also been reported to inhibit dissociation of pCRP to mCRP on the surface of circulating microparticles isolated from the blood of patients with myocardial infarction (69). Pretreatment with bis-PC abolished the increase in infarct size and cardiac dysfunction produced by injection of human pCRP in a rat model of myocardial infarction (72). A controversy exists whether rat CRP can activate rat complement and whether rat factor H, the native complement-control protein, could interact with human CRP (116, 117). Hence, the translational implication of these observations remains to be clarified. Binding of CRP decamers to Fcγ receptors or possible deposition of large CRP complexes might trigger immune reactions, thereby limiting the therapeutic use of bis-PC. This compound is apparently no longer being considered for clinical development [http://pentraxin.word-press.com/rd-programs/].

Considering the role of native CRP in host defense, therapies aimed at reducing serum pCRP level would likely impair defense mechanisms and predispose to infection. Thus, an attractive alternative approach is to selectively block expression of pro-inflammatory properties “hidden” in pCRP without interfering with its protective functions. As a proof of this concept, Zeller et al. (65) developed a low molecular weight monovalent compound C10M [3-(dibutyl amino)propyl) phosphonic acid]. C10M binds to the phosphocholine binding pocket of pCRP and prevents pCRP binding to phosphocholine residues exposed on the surface of activated or damaged cells or microvesicles, and subsequently the formation of pCRP*/mCRP (65). Apart from the occupied phosphocholine binding pocket, the B-face remains accessible to other ligands, including misfolded or aggregated proteins or proteins whose secondary structure is predominantly β-sheet (56) as well as neutrophils. Consistently, C10M inhibited pCRP*/mCRP-stimulated activation of monocytes and neutrophils, extrusion of neutrophil extracellular traps (NETs), monocyte adhesion to activated endothelial cells, and formation of platelet-monocyte aggregates (65). C10M reduction of pCRP*/mCRP-triggered, presumably ROS-dependent NET release (i.e. *via* the suicidal pathway) could contribute to preventing NET-mediated tissue damage under pathological conditions (54, 118). Importantly, C10M did not impair antibacterial host defense as evidenced by unaltered pCRP opsonization-mediated phagocytosis of bacteria by monocytes and neutrophils (65). Furthermore, C10M efficiently suppressed tissue deposition of human pCRP*/mCRP and monocyte accumulation within the affected organs in murine models of renal ischemia-reperfusion and allograft rejection of hindlimb transplants (65). Consistently, C10M significantly improved renal function following ischemia-reperfusion, and prevented premature loss of allograft transplants driven by human pCRP. While these findings would indicate the functional importance of mCRP, further studies are needed to distinguish the effects of CRP antagonists on endogenous CRP and injected human CRP in these and other experimental models.

## Concluding remarks

CRP is a well-established biomarker of inflammation and much written about its association with disease state. Circulating hs-CRP may identify patients at risk, predict disease progression and outcome, and guide therapy in a context-dependent manner. Nevertheless, since even strong associations do not establish causality, further studies are clearly warranted to elucidate its potential pathogenic roles. Activation-induced conformational changes in pCRP would unmask “hidden” pro-inflammatory properties as opposed to the largely protective role of pCRP. CRP lowering strategies yielded promising, but often inconclusive data on altering disease progression. Development of CRP antagonists, and in particular recent development of a phosphocholine mimetic that binds to pCRP and inhibits conformation change-mediated expression of pro-inflammatory activities without impairing pCRP’s defense function, should facilitate future investigations into the full spectrum of the roles of CRP isomers in inflammatory pathologies. This approach has the potential of opening a novel therapeutic avenue for preventing or limiting the deleterious actions attributed to CRP.

## Author contributions

All authors contributed to the article and approved the submitted work.
